# Primary Omental Lipoma in a Child: A Case Report and Literature Review

**DOI:** 10.3389/fped.2021.820845

**Published:** 2022-01-28

**Authors:** Qiang Yuan, Xufei Duan, Xueqiang Yan

**Affiliations:** Department of Pediatric Surgery, Wuhan Children's Hospital (Wuhan Maternal and Child Healthcare Hospital), Tongji Medical College, Huazhong University of Science and Technology, Wuhan, China

**Keywords:** primary omental lipoma, laparoscopy, child, US, resect

## Abstract

**Background:**

Lipoma is a common benign tumor derived from adipose tissue, with an incidence of nearly 10%. It is the most common mesenchymal tumor throughout the body. However, the pathogenesis of lipoma is not clear yet, and the increased incidence is attributable to obesity, elevated serum cholesterol, diabetes, trauma, radiation, familial predisposition, and chromosome. Primary omental tumor is a rare lipoma occurring in the greater omentum, most of which is reported in the form of clinical case reports. Nevertheless, primary omental tumor is even rarer in children. To date, there have been few reports of clinical cases.

**Case Presentation:**

We report a rare case of primary omental lipoma in a 6-year-old boy. After an accidental fall, a CT scan found that he had a tumor in the left upper abdomen. He had no history of abdominal pain, abdominal mass, vomiting, etc. The boy was admitted to the hospital within 3 days, and was diagnosed with an intra-abdominal tumor. After admission, abdominal ultrasound and enhanced CT showed a 71 ×40 ×60 mm mass in the left middle abdomen, which was considered a lipoma. There was no abnormality in tumor markers. Through laparoscopic surgery, intraoperative exploration revealed that the tumor was located in the left mid-upper abdomen, and was yellow, solid, soft, and isolated. The intraoperative diagnosis was an omental lipoma. We used an ultrasonic knife to resect the omentum close to the base of the tumor. The tumor was completely resected, put in a retrieval bag and sealed. Finally, the left and right sides of the umbilical incision were extended to take out the tumor tissue. The child received liquid food 6 h after the operation and was discharged 3 days later. The postoperative pathological diagnosis was an omental lipoma. He was seen at follow-up 3 months after discharge and had no complaints, an abnominal ultrasound showed no tumor recurrence.

**Conclusion:**

Primary omental lipoma in children is a rare benign tumor of the omentum. Its etiology and pathology are not clear. US, CT, and MRI can facilitate clinical diagnosis and preoperative evaluation. Laparoscopic surgery is an effective treatment, and the prognosis of children is favorable. This case is beneficial to improve the clinical knowledge of pediatric surgeons about this rare disease.

## Introduction

Primary omental lipoma is a rare benign tumor of the omentum. The pathogenesis is not clear, and there is no statistical data on its incidence. This lipoma in children is even rarer, most of which are reported in the form of clinical case reports. This study reports the diagnosis and treatment of a 6-year-old boy with primary omental lipoma. Meanwhile, it discusses the clinical characteristics as well as methods of diagnosis and treatment of primary omental lipoma based on previous literature data. This study was approved by the Research and Ethics Committee of our institution, and written informed consent was obtained from the patient's family.

## Case Presentation

**Figure 1:**
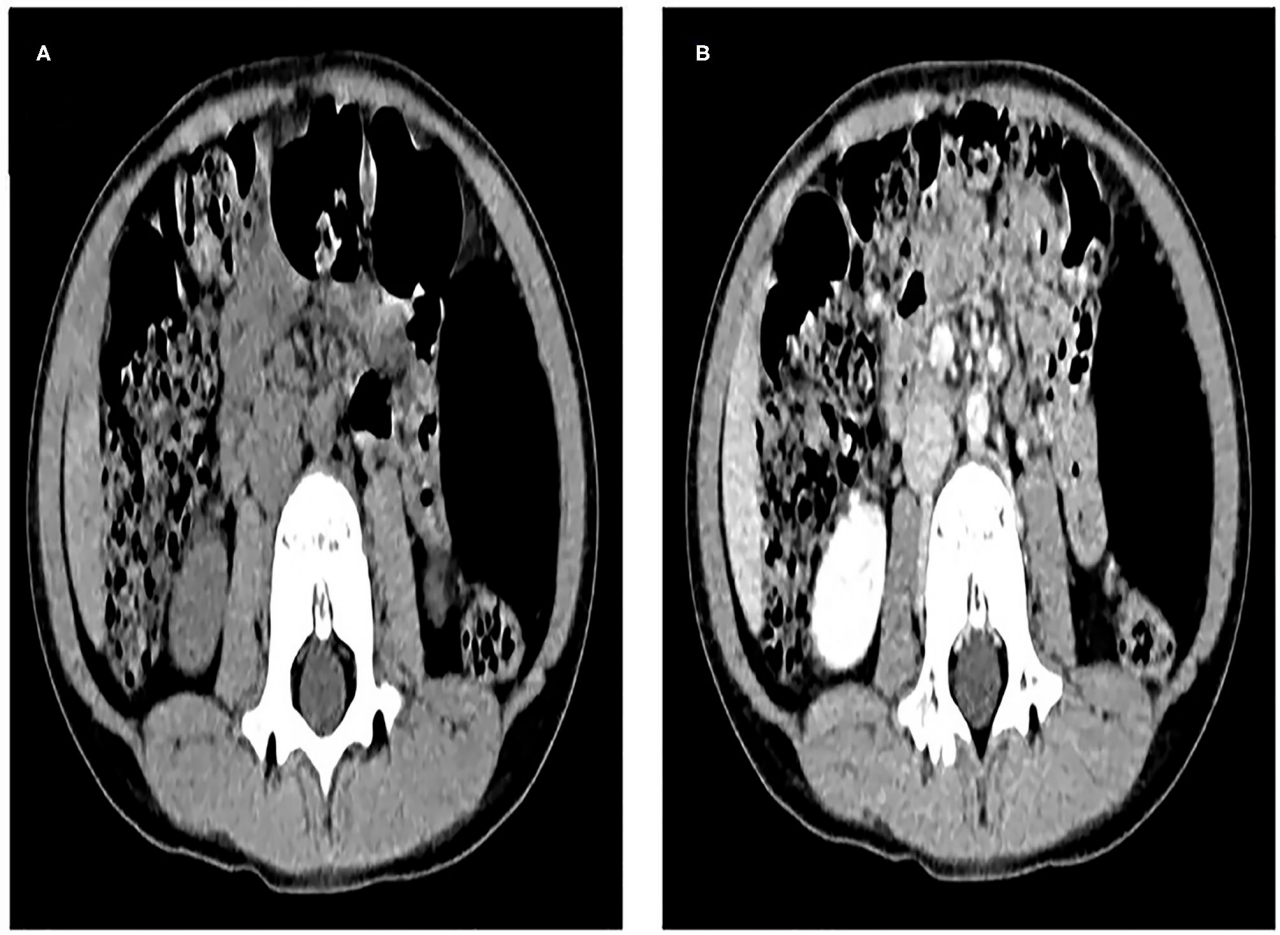
**(A)**, Computed tomography (CT) scan shows an elliptical, very low-density mass in the left middle abdomen, which is wrapped in the left abdominal cavity, and the surrounding intestine is slightly compressed and displaced. **(B)**, The tumor was not enhanced on enhanced CT scan, and its blood supply arteries were not shown.

A 6-year-old male child was found to have a tumor in the left mid-upper abdomen on a CT examination of his abdomen after an accidental fall. The boy had no history of abdominal pain, abdominal mass, and vomiting. He was admitted to the hospital with an abdominal tumor within 3 days. The initial diagnosis was an intraperitoneal tumor. After admission, laboratory tests showed no abnormality in blood cell count, biochemical analysis, and tumor markers. Abdominal ultrasound and enhanced CT showed a mass in the left middle abdomen, which size was 71 ×40 ×60 mm. Intra-abdominal lipoma should be considered, and the blood-supplying vessels were derived from branches of the omental artery (Figures 1A,B).

**Figure 2:**
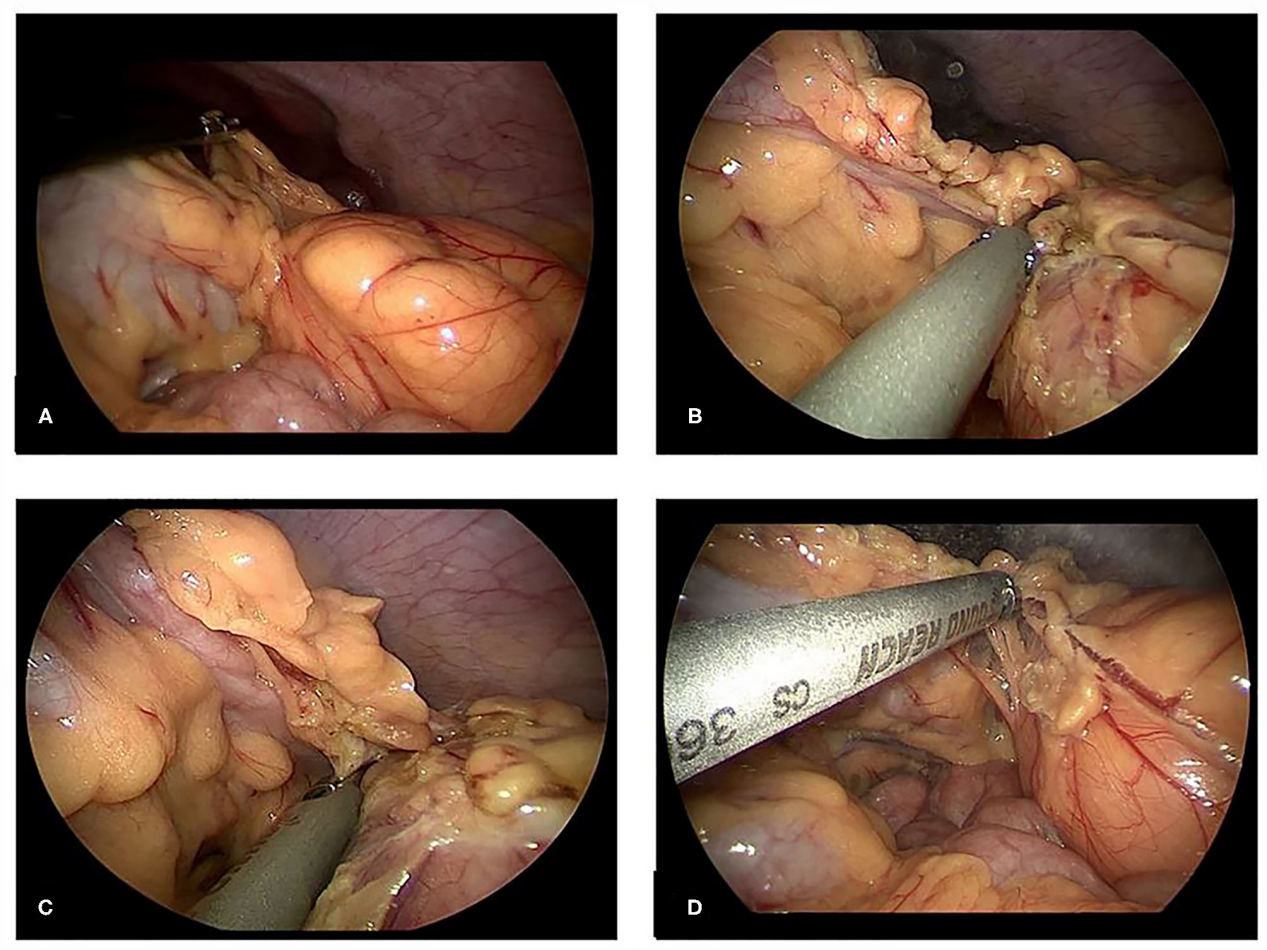
**(A)**, Laparoscopic examination of the left mid-upper abdominal tumor, which was yellow, solid, isolated, derived from the greater omentum, without torsion. **(B,C)**, Ultrasonic knife separates the omentum and the tumor. **(D)**, Ultrasonic scalpel coagulates the tumor blood supply artery.

We performed laparoscopy surgery on the child: three incisions for 5 mm trocars were made on the insufflated abdomen respectively at the left and right sides of the umbilicus and at the anti-Mc Burney's point. Intraoperative laparoscopic exploration revealed a tumor in the left part of middle and upper abdomen, with a size of about 70 ×60 ×50 mm. It was yellow, solid, soft, isolated, and its base was derived from the greater omentum without torsion (Figure 2A). Preoperative and intraoperative assessments revealed that the tumor could be successfully and completely resected by laparoscopic surgery. The ultrasound knife cut off the greater omentum and the blood-supplying vessels at the base of the tumor. The tumor was completely removed and was placed in the specimen retrieval bag which was then closed. The ultrasound knife coagulated the residual end of the greater omentum to stop bleeding. No other abnormality was found in the abdominal cavity below the umbilicus to take out the tumor tissue completely (Figures 2B–D).

**Figure 3:**
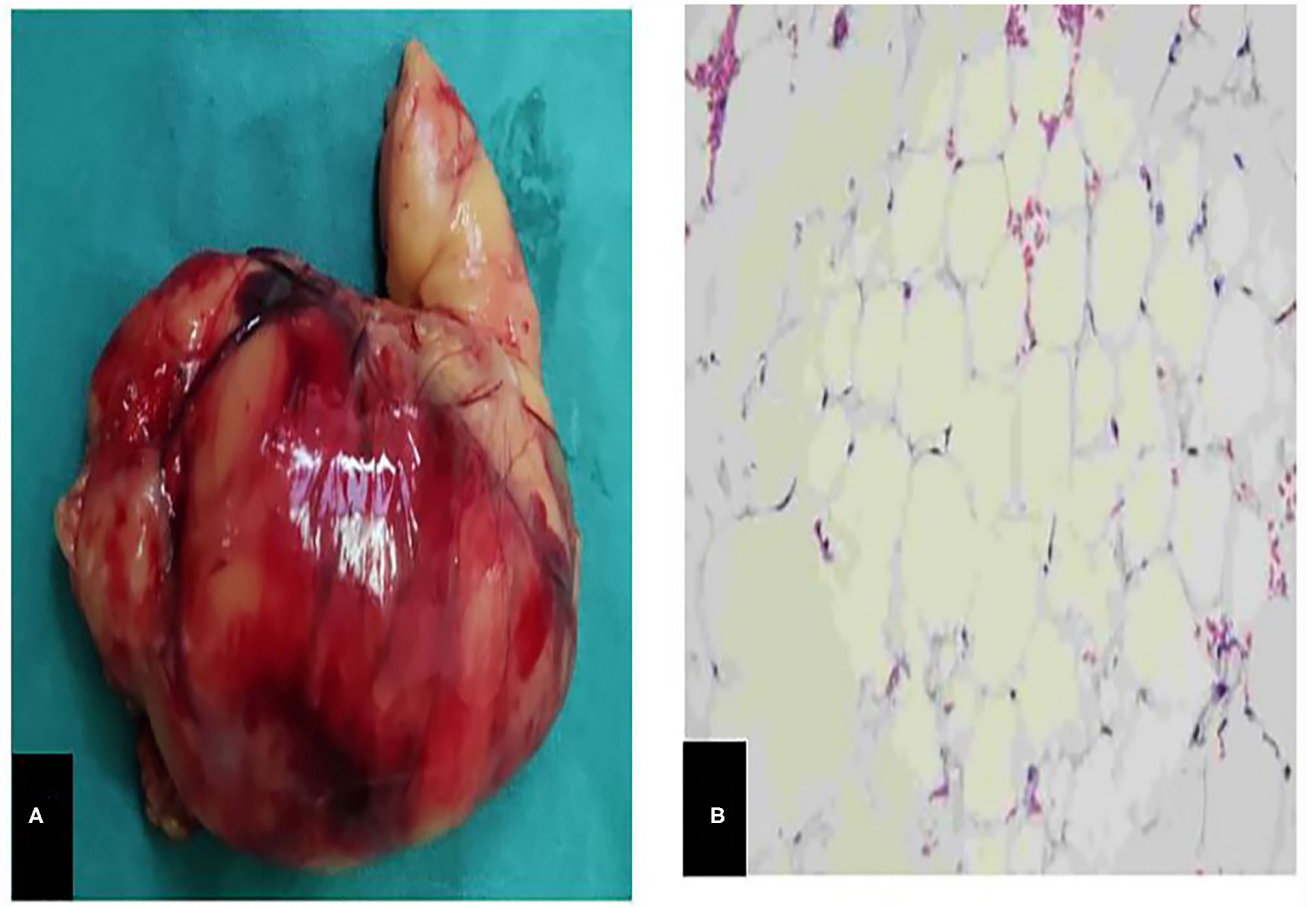
**(A)**, A piece of yellow tissue can be seen with the naked eye, 295g in weight, about 80mm* 55mm * 30mm in size, with intact capsule, solid and soft. **(B)**, Microscopically, the tumor is composed of mature adipocytes without atypia.

The operation time was 27 min. The weight of the excised tumor tissue was 295 g, size of about 80 ×55 ×30 mm with; the tumor capsule intact; and the cut surface is grayish-yellow and soft (Figure 3A). Microscopic examination showed that the tumor was composed of mature adipocytes without atypia (Figure 3B); and the diagnosis was of a benign omental lipoma. The patient was discharged 3 days postoperatively without further complication, follow-up abdominal ultrasonography after 3 months showed no recurrence of lipoma.

Pubmed, Springer Link, CNKI, and Wanfang databases were searched before June 2021 to retrieve the related literature of omental lipoma, a total of 12 clinical reports of primary omental lipoma in children were retrieved in the previous literature (Table 1).

**Table 1:** Cases of children reported to have omental lipoma found in the available literature.

**Case**	**Authors**	**Year**	**Age**	**Sex**	**Clinical presentation**	**Weigh (g)**	**Size (cm)**	**Treatment**
1	Haller	1978	3y	F	NR	NR	8*5*4	Excision
2	Giubilei	1980	8y	M	NR	NR	18*15*8	Excision
3	Joulak	1998	3y	M	NR	NR	13*9*6	Excision
4	Barauskas	2004	8y	F	NR	720	11*10*8	Excision
5	Luo	2005	11m	M	Abdominal distention	1820	21*15*12	Excision
6	Srinivasan	2009	9m	NR	NR	1500	NR	Excision
7	Abubakar	2009	13y	F	NR	12300	34*26*22	Excision
8	Chaudhary	2011	2y	M	NR	NR	NR	Excision
9	Cascini	2012	19m	M	Abdominal distention	1185	22*18*8	Excision
10	Cascini	2012	7y	F	Abdominal distention	2070	25*22*10	Excision
11	Cascini	2012	10y	F	Abdominal pain	1370	18*12*10	Excision
12	Kinjo	2014	5y	F	NR	335	8*6*3	Excision
13	Present case (2021)	2021	6y	M	NR	295	8*5.5*3	Excision

*NR, not reported; y, years; m, moths*.

## Discussion And Conclusions

Lipoma is a common benign tumor derived from adipose tissue, with an incidence of nearly 10% (1). It is the most common mesenchymal tumor throughout the body. However, the pathogenesis of lipoma is not clear yet, and the increased incidence is attributable to obesity, elevated serum cholesterol, diabetes, trauma, radiation, familial predisposition, and chromosome abnormality (2, 3). The omentum is a double-layer membrane composed of peritoneum and adipose tissue, which is attached to the greater curvature of the stomach and transverse colon. It covers the abdominal organs in the abdominal cavity in the shape of a skirt, including blood vessels, nerves, lymphatic vessels, and connective tissue (4, 5). Primary omental lipoma is very rare, and most of which is described in the form of case reports. A variety of pathologies have been reported in the clinic, such as leiomyosarcoma, fibrosarcoma, hemangiopericytoma, liposarcoma, leiomyoma, lipoma, fibroma, and mesothelioma tumors. Lipoma may be the rarest of all the above forms, with an extremely low incidence, accounting for about 7–9% of omental tumors (6, 7).

Review-of the retrieved literature of 12 cases of primary omental lipoma in children, found that children with omental lipoma are usually asymptomatic and are discovered incidentally during medical examination, trauma, or abdominal diseases. The main symptoms of these children include abdominal pain, abdominal distention, and abdominal mass. Nausea, weight loss, and intraperitoneal hemorrhage occurred occasionally, and a few children were admitted to the emergency department due to torsion of the omentum. It has been reported that adult patients were hospitalized due to intussusception and intestinal obstruction (8, 9).

Common imaging methods for diagnosing omental tumors include ultrasound, CT, and MRI. It is clinically difficult to discriminate benign and malignant omental tumors. The differential diagnosis of omental lipoma includes lymphangioma, lymphoma, duplication of the digestive tract, and neuroblastoma. However, the main differential diagnosis is lipoblastoma (10). Omental lipoma is easily found by ultrasound examination. Ultrasound can show that the tumor has heterogeneous echo and abundant blood flow signals, it also reveals the size of the tumor. Smaller omental lipomas are sometimes misdiagnosed as normal mesenteric fat (11). Ultrasound elastography is a non-invasive imaging technology based on different tissue hardness which have been developed in recent years. It can measure the elasticity of the tissue, according to the various performance of the tissue under different external pressure, then to distinguish benign and malignant lesions. Zhang et al. (12) explored whether ultrasound elastography is effective in the diagnosis of benign and malignant omentum thickening. The results showed that the elasticity score of the malignant omentum thickening group was higher than that of the benign omentum thickening group (*P* <0.01). In addition, ultrasound-guided percutaneous needle biopsy has been a commonly used method for the diagnosis of intra-abdominal lesions, such as liver, kidney, pancreas lesions, and other solid organs, but it is not commonly used for peritoneal and omental lesions. It may cause controversy by seeding tumor cells along the needle path (13). CT examination can show clear, uniform, and low-density intra-abdominal mass, which may have fibrous partitions and a few calcification points inside. Enhanced CT helps to assess the relationship between the tumor and surrounding organs, and also helps to identify the blood-supplying arteries of the tumor (14). MRI has high specificity for the diagnosis of simple lipoma and can distinguish it from well-differentiated liposarcoma. By using an MRI pulse sequence, lipomas show signal intensity similar to that of fat on high T1 signal and intermediate T2 signal. If there are thicker intervals inside the tumor, nodules, and non-fat-like masses, the proportion of fat in the lesion will decrease. It suggests the diagnosis of liposarcoma (15, 16).

Laparoscopic exploration is an important method to confirm that lipomas originate from the greater omentum during the operation, and surgical resection is an effective treatment for the lipoma of the greater omentum. The tumor can be completely resected under laparoscopy.When a patient is admitted to the hospital due to torsion of the greater omentum, laparoscopic exploration can be performed in the emergency department, both laparoscopic tumor resection and the greater omentum resection can be performed at the same time. According to previous reports in the literature, the recurrence rate after resection is <5%, mainly due to incomplete resection (17).

In conclusion, we reported a rare case of primary omental lipoma in children. By reviewing the previous literature, we found that primary omental lipoma in children is very rare. Preoperative ultrasound and enhanced CT can determine intra-abdominal lipoma, which can also help to identify the origin of the tumor's blood vessels. Intraoperative exploration of laparoscopic surgery can confirm that the lipoma originates from the greater omentum. The lipoma can be completely resected under laparoscopic surgery. If combined with torsion of the greater omentum, partial resection of the greater omentum can be performed at the same time. Laparoscopic surgery is minimally invasive and generally without complications. The prognosis of the child is good.

## Data Availability Statement

The original contributions presented in the study are included in the article/supplementary material, further inquiries can be directed to the corresponding author.

## Ethics Statement

The studies involving human participants were reviewed and approved by Wuhan Children's Hospital (Wuhan Maternal and Child Healthcare Hospital), Tongji Medical College, Huazhong University of Science and Technology. The patients/participants provided their written informed consent to participate in this study. Written informed consent was obtained from the individual(s), and minor(s)' legal guardian/next of kin, for the publication of any potentially identifiable images or data included in this article.

## Author Contributions

QY and XY designed the study, collected the clinical data, performed the statistical analysis, participated in the operation, and drafted the manuscript. XD participated in the operation and revised the article. All authors read and approved the final manuscript.

## Conflict of Interest

The authors declare that the research was conducted in the absence of any commercial or financial relationships that could be construed as a potential conflict of interest.

## Publisher's Note

All claims expressed in this article are solely those of the authors and do not necessarily represent those of their affiliated organizations, or those of the publisher, the editors and the reviewers. Any product that may be evaluated in this article, or claim that may be made by its manufacturer, is not guaranteed or endorsed by the publisher.
